# Hepatitis B Virus S Protein Enhances Sperm Apoptosis and Reduces Sperm Fertilizing Capacity In Vitro

**DOI:** 10.1371/journal.pone.0068688

**Published:** 2013-07-16

**Authors:** JiHua Huang, Ying Zhong, XiaoWu Fang, QingDong Xie, XiangJin Kang, RiRan Wu, FangZheng Li, XiaoQin Xu, Hui Lu, Lan Xu, TianHua Huang

**Affiliations:** 1 Guangdong Provincial Key Laboratory of Infectious Diseases and Molecular Immunopathology, Research Center for Reproductive Medicine, Shantou University Medical College, Shantou, Guangdong, China; 2 Center for Reproductive Medicine, Chengdu Jingjiang Hospital for Maternal and Child Health Care, Chengdu, Sichuan, China; 3 Center for Reproductive Medicine, Boai Hospital of Zhongshan, Zhongshan, Guangdong, China; Clermont-Ferrand Univ., France

## Abstract

**Objective:**

Studying the impact of Hepatitis B virus S protein (HBs) on early apoptotic events in human spermatozoa and sperm fertilizing capacity.

**Methodology/Principal Findings:**

Spermatozoa were exposed to HBs (0, 25, 50, 100 µg/ml) for 3 h, and then fluo-4 AM calcium assay, Calcein/Co^2+^ assay, protein extraction and ELISA, ADP/ATP ratio assay, sperm motility and hyperactivation and sperm-zona pellucida (ZP) binding and ZP-induced acrosome reaction (ZPIAR) tests were performed. The results showed that in the spermatozoa, with increasing concentration of HBs, (1) average cytosolic free Ca^2+^ concentration ([Ca^2+^]_i_) rose; (2) fluorescence intensity of Cal-AM declined; (3) average levels of cytochrome c decreased in mitochondrial fraction and increased in cytosolic fraction; (4) ADP/ATP ratios rose; (5) average rates of total motility and mean hyperactivation declined; (6) average rate of ZPIAR declined. In the above groups the effects of HBs exhibited dose dependency. However, there was no significant difference in the number of sperms bound to ZP between the control and all test groups.

**Conclusion:**

HBs could induce early events in the apoptotic cascade in human spermatozoa, such as elevation of [Ca^2+^]_i_, opening of mitochondrial permeability transition pore (MPTP), release of cytochrome c (cyt c) and increase of ADP/ATP ratio, but exerted a negative impact on sperm fertilizing capacity.

## Introduction

Hepatitis B is a potentially life-threatening liver infection caused by hepatitis B virus (HBV). It is a major global health problem and can cause chronic liver disease and puts people at high risk of death from cirrhosis of the liver and liver cancer. Worldwide, an estimated two billion people have been infected with HBV and more than 240 million have chronic (long-term) liver infections. About 600 000 people die every year due to the acute or chronic consequences of hepatitis B [1]. Therefore, studies on the relationship between HBV infection and human health are very important. In recent literature, it has been showed that men infected with hepatitis B may have low fertility, which attracted attention of the researchers.

The subviral particles of HBV, which predominantly comprise HBs, are produced in vast excess over HBV virions into the circulation where concentrations reach 50–300 µg/ml [2]. It has been demonstrated that HBV is able not only to pass through the blood-testis barrier and enter male germ cells but also integrate into their genome to cause male infertility by damaging spermatozoa [3]–[6]. Some reported that HBV has a deleterious effect on sperm motility *in vivo* and that the couples whose male partner is infected have a higher risk of low fertilization rate after *in vitro* fertilization [7]. Although viral infection can affect male fertility, to date, however, only scant information is available about the influence of HBV infection on sperm function and its exact molecular mechanisms.

Recent publications reported that apoptosis may play a major role in causing diseases related to male infertility [8]. An altered apoptosis process has been found to be closely associated with male infertility and with sperm quality such as motility, viability and sperm defects [9], [10]. Furthermore, viral infection can actively elicit apoptosis, and higher proportion of apoptotic and necrotic spermatozoa in the patients with chronic HBV infection has been documented [11]. Our previous study showed that co-incubation of human sperms with HBs caused a series of apoptotic events including loss of mitochondrial membrane potential (MMP), generation of reactive oxygen species (ROS), lipid peroxidation, reduction of total antioxidant capacity, externalization of phosphatidylserine (PS), activation of caspases, and DNA fragmentation, resulting in reduced sperm motility and loss of sperm membrane integrity, and causing sperm dysfunction, diminished fertility, and sperm death [12], [13]. In the present study, we investigated the effects of HBs exposure on the early apoptotic events in human spermatozoa, including [Ca^2+^]*_i_*, modulation of MPTP, level of cyt c and ADP/ATP ratio, and on sperm fertilizing capacity, such as sperm motility parameters, sperm-ZP binding and the ZP-induced AR (ZPIAR) of ZP-bound sperms.

## Results

### Changes in [Ca^2+^]***_i_***

**Figure 1:**
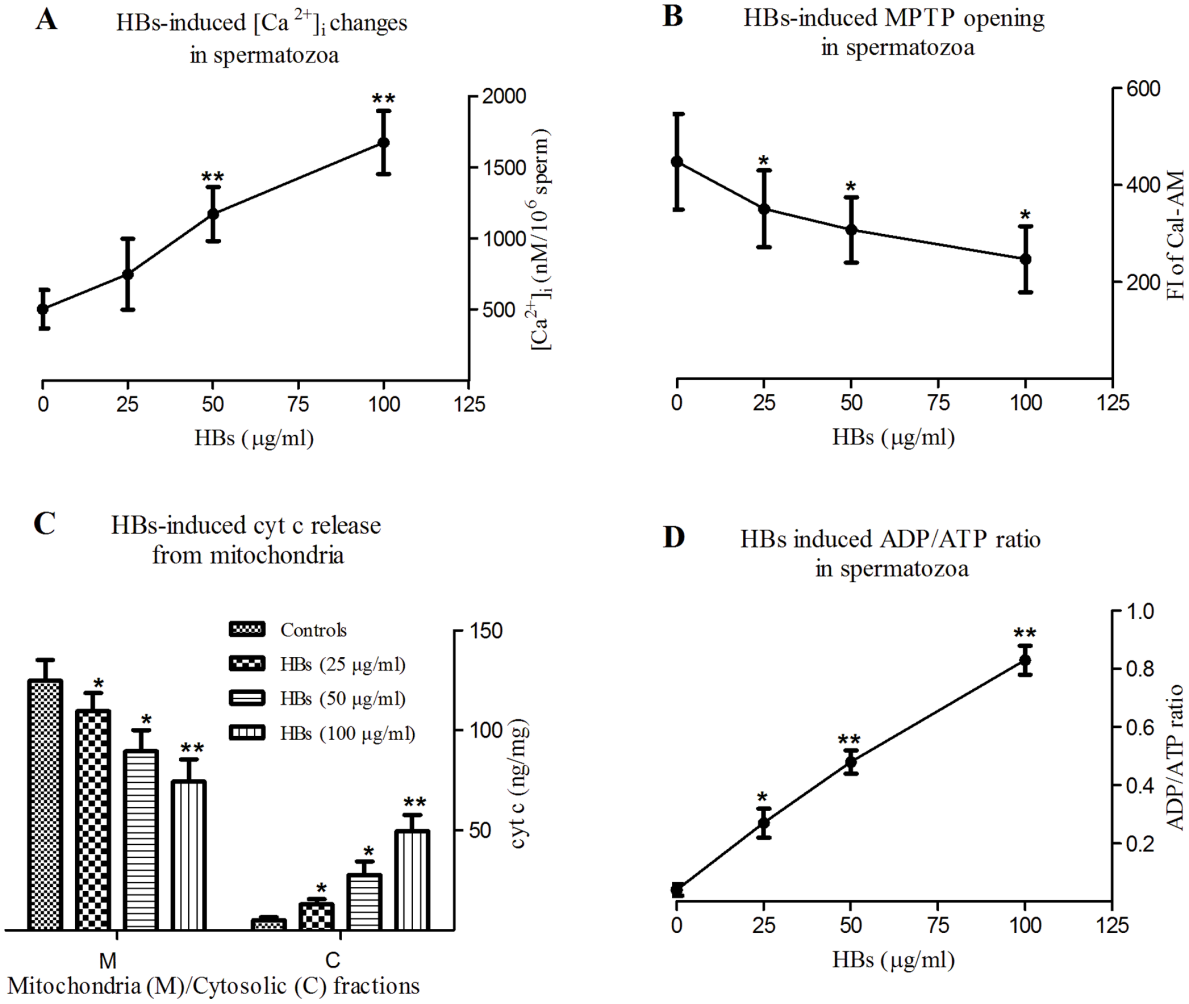
HBs induced early apoptotic events in human spermatozoa. A: The average [Ca^2+^]_i_ rose with increasing concentrations of HBs. There are marked significant differences in the average [Ca^2+^]_ i_ in 50 µg/ml and 100 µg/ml HBs groups when compared with that in the control (P<0.01). The high levels of [Ca^2+^]_ i_ induced by HBs would be a signal to trigger apoptosis. B: In contrast, FI of Cal-AM declined with increasing concentrations of HBs. There are significant differences in FI between the control and test groups (P<0.05). This suggested that MPTP opening has occurred, and the extent of pore opening increased with increasing concentrations of HBs. C: the levels of cyt c in mitochondria fractions declined with increasing concentrations of HBs, in parallel, the levels of cyt c in cytosolic fractions rose with increasing concentrations of HBs. There are significant differences in levels of cyt c in all test groups as compared to that in control (P<0.05 or P<0.01). This suggested that cyt c was released from mitochondria to cytoplasm through the MPTP. D: The ADP/ATP ratio rose with increasing concentrations of HBs. There are significant differences in ADP/ATP ratio between the control and all test groups (P<0.01). It indicated that spermatozoa underwent apoptosis induced by HBs. In all above events, effects of HBs exposure on spermatozoa showed dose dependency.

The results are shown in Table 1, Fig. 1A. The average [Ca^2+^]*_i_* were 502.32±135.25, 748.06±249.27, 1171.11±189.12 and 1673.94±223.02 nmol/10^6^ sperm in 0, 25, 50, 100 µg/ml HBs-exposed groups, respectively. The average [Ca^2+^]_i_ rose with increasing concentration of HBs. A marked significant increases in average [Ca^2+^]_i_ were observed after 3 h exposure to 50 and 100 µg/ml of HBs as compared to that in control (P<0.01).

**Table 1:** Effects of HBs exposure on early apoptotic events in human spermatozoa.

Related Parameters	Controls	HBs concentration		
		25 µg/ml	50 µg/ml	100 µg/ml
[Ca^2+^]i (nM/10^6^ sperm)	502.32±135.25	748.06±249.27	1171.11±189.12**	1673.94±223.02**
MPTP (FI of Cal-AM)	433.59±119.47	356.06±96.87*	291.64±80.31*	214.66±73.7*
cyt c (ng/mg) in mitochondria	124.95±10.47	109.69±9.17*	89.58±10.78*	74.44±11.16**
cyt c (ng/mg) in cytosol	5.04±1.52	13.01±2.5*	27.73±6.75*	49.66±8.12**
ADP/ATP ratio	0.04±0.02	0.27±0.05*	0.48±0.04**	0.83±0.05**

Note: [Ca^2+^]i: concentration of intracellular calcium; MPTP: mitochondrial permeability transition pore; FI: fluorescent intensity; cyt c: cytochrome c; ADP: Adenosine diphosphate; ATP: adenosine triphosphate. These data are representatives of five independent experimental replications (five individuals). The average values are expressed as mean ± SD. A paired t test was conducted to evaluate the impact of exposures of various concentration of HBs. *P<0.05; **P<0.01.

### Assessment of MPTP

**Figure 2:**
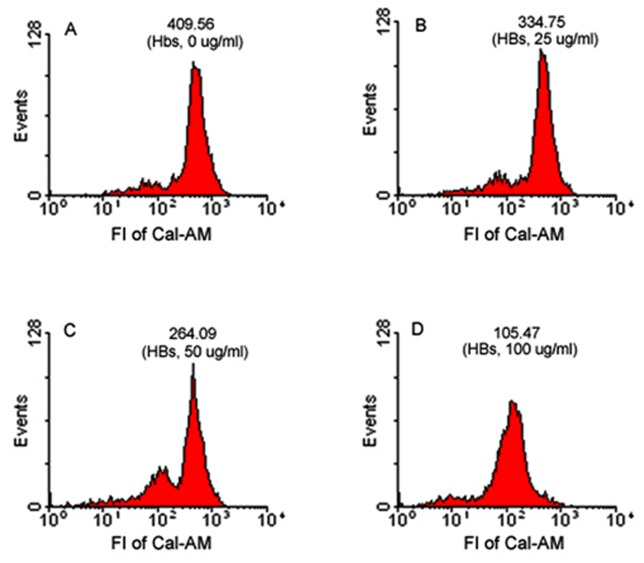
HBs induced MPTP activation in human spermatozoa. The histograms of the number of events (Y-axis) versus the FI (X-axis) in the individual experiment showed that FI of Cal-AM in spermatozoa declined with increasing concentration of HBs, and the number of spermatozoa showing diminished FI rose with increasing concentration of HBs. The change in FI Cal-AM in spermatozoa between panels indicates the continuous activation of MPTP.

In the individual experiment, the fluorescent intensities (FI) of Cal-AM in spermatozoa were 409.56, 334.75, 264.09 and 105.47 in 0, 25, 50, 100 µg/ml HBs-exposed groups, respectively (Fig. 2). In five experiments, the average FI of Cal-AM in spermatozoa were 433.59±119.47, 356.06±96.87, 291.64±80.31 and 214.66±73.7 in 0, 25, 50, 100 µg/ml HBs-exposed groups, respectively (Table 1, Fig. 1B). The FI of Cal-AM in spermatozoa, either its single value or its average value, declined with increasing concentration of HBs (Fig. 1 and 2), and the number of spermatozoa showing diminished fluorescence rose with increasing concentration of HBs (Fig. 2). There were significant differences in average FI of Cal-AM in all test groups as compared to that in control (P<0.05).

### Assessment of Cyt c Release from Mitochondria

The levels of cyt c in mitochondrial fraction were 124.95±10.47, 109.69±9.17, 89.58±10.78, 74.44±11.16 ng/mg in 0, 25, 50, 100 µg/ml HBs-exposed groups, respectively. In parallel, the levels of cyt c in cytosolic fraction were 5.04±1.52, 13.01±2.5, 27.73±6.75 and 49.66±8.12 ng/mg in the above groups, respectively. The levels of cyt c decreased in mitochondrial fraction and increased in cytosolic fraction with increasing concentration of HBs (Table 1, Fig. 1C). There were significant differences in average levels of cyt c in all test groups when compared with that in control (P<0.05 and P<0.01).

### The ADP/ATP Ratio

The ADP/ATP ratios in spermatozoa were 0.04±0.02, 0.27±0.05, 0.48±0.04 and 0.83±0.05 in 0, 25, 50, 100 µg/ml HBs-exposed groups, respectively, and rose with increasing concentrations of HBs (Table 1, Fig. 1D). There were significant differences in ADP/ATP ratio in all test groups as compared to that in control (P<0.01).

### Sperm Motility Parameters

**Figure 3:**
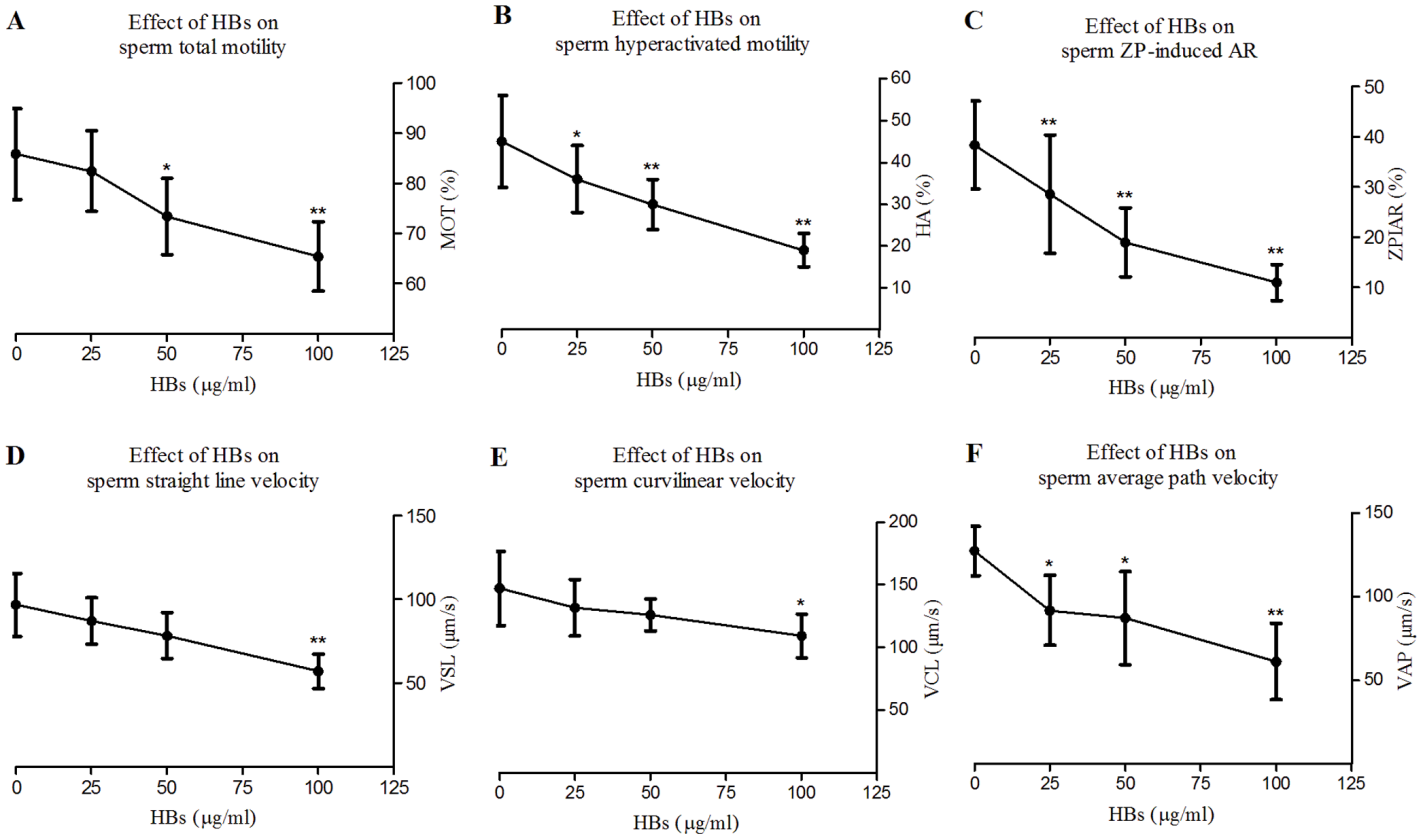
Effects of HBs exposure on sperm fertilizing capacity. A and B: The average rates of MOT and the mean HA rates declined with increasing concentrations of HBs. There were significant differences in the average rates of MOT in 50 and 100 mg/ml HBs groups and in the mean HA rates in all test groups when compared with those in the controls (P<0.05 or P<0.01). C: The average rates (%) of ZPIAR declined with increasing concentrations of HBs. There were marked significant differences in ZPIAR rates in all test groups as compared to that in control (P<0.01). D–F: The sperm velocities including VSL, VCL and VAP declined with increasing concentrations of HBs. There were significant differences in VSL and VCL in 100 mg/ml HBs group and in VAP in all test groups when compared with those in controls. The above results showed that HBs exposure could affect sperm fertilizing capacity.

The average rates (%) of total motility (MOT) were 85.80±4.04, 82.40±3.42, 73.40±3.59 and 65.40±3.09 in 0, 25, 50, 100 µg/ml HBs-exposed groups, respectively, and the mean hyperactivation (HA) rates (%) were 45.00±11.00, 36.00±8.00, 30.00±6.00 and 19.00±4.00 in the above groups, respectively. The average rate of MOT and the mean HA rate declined with increasing concentrations of HBs (Fig. 3A and 3B). There were significant differences in the average rates of MOT in 50 and 100 µg/ml HBs-exposed groups and in the mean HA rates in all test groups when compared with those in controls (P<0.05 and P<0.01). The sperm velocities including VSL, VCL and VAP in the above groups declined with increasing concentrations of HBs. There were significant differences in VSL and VCL in 100 µg/ml HBs group and in VAP in all test groups when compared with those in controls (Fig. 3D, 3E and 3F). The significant differences in other parameters including concentration (CON), progressive motility (PMOT), straightness (STR), linearity (LIN), amplitude of lateral head displacement (ALH) and beat cross frequency (BCF) among the above groups have not been observed.

### Sperm-ZP Binding

The medians in number of sperms bound to ZP were 100 in the 0, 25, 50, 100 µg/ml HBs-exposed groups, and their quartile deviations were 30, 25, 30, 30 in the above groups, respectively. There was no significant difference in number of sperms bound to ZP between any of the four groups (P>0.05).

### Assessment of the AR of Sperms Bound to the ZP

The average rates (%) of ZPIAR were 38.36±8.73, 28.57±11.76, 18.99±6.84 and 10.98±3.58 in 0, 25, 50, 100 µg/ml HBs-exposed groups, respectively, and declined with increasing concentrations of HBs (Fig. 3C). There were marked significant differences in ZPIAR rates in all test groups as compared to that in control (P<0.01).

## Discussion

### HBs Induced Early Apoptotic Events in Human Spermatozoa

Recent publications have suggested that mitochondria of human spermatozoa are preferentially susceptible to apoptosis in response to various types of intracellular stress [14]. One of the responsible signals for initiating the process of apoptosis is a sustained increase in [Ca^2+^]_i_ [8]. In the present study, the average [Ca^2+^]_i_ in spermatozoa rose with increasing concentration of HBs. Significant increases in average [Ca^2+^]_i_ were observed after 3 h exposure to 50 and 100 µg/ml of HBs as compared to that in control (P<0.01). This suggested that HBs exposure induced the entry of calcium ions into cells through the plasma membrane or release of Ca^2+^ from internal stores, leading to the elevation of [Ca^2+^]_i_ in spermatozoa. [Ca^2+^]_i_ was one of the signals initiating the process of apoptosis in this study.

[Ca^2+^]_i_ homeostasis is regulated by mitochondria, especially by MPTP, which is a nonspecific channel formed by components from the inner and outer mitochondrial membranes. Calcium enters the matrix via the mitochondrial Ca^2+^ uniporter and exits by exchange with Na^+^ on the Na^+/^Ca^2+^ antiporter. Normally, MPTP may “flicker” to release excess matrix Ca^2+^ to maintain [Ca^2+^]_i_ homeostasis. However, when cells become pathologically overloaded with calcium, the MPTP opens irreversibly. Continuous MPTP activation causes mitochondrial Ca^2+^ overload, leading to mitochondrial dysfunction [15]. In the present study, the FI of Cal-AM in the spermatozoa declined with increasing concentration of HBs, and the number of spermatozoa showing diminished fluorescence rose with increasing concentration of HBs. There were significant differences in average FI of Cal-AM in all test groups as compared to that in control (P<0.05). Our results also revealed that HBs increased MPTP opening and the number of spermatozoa with pore opening in a dose-dependent manner. It has been reported that the sensitivity of MPTP to [Ca^2+^] is greatly enhanced by oxidative stress, phosphate and adenine nucleotide depletion [16], [17]. Our previous study has detected that HBs could induce oxidative stress in spermatozoa [13]. Therefore, HBs-induced MPTP opening could result from the synergistic effects of Ca^2+^ overload, ATP depletion and oxidative stress in this study.

Cyt c is a component of the electron transport chain in mitochondria and involved in initiation and is an intermediate of apoptosis. Activated MPTP releases cyt c from the intermembrane space into the cytoplasm. In the present study the average levels of cyt c declined in mitochondria fraction and rose in cytosolic fraction with increasing concentration of HBs. There were significant differences in average levels of cyt c in all test groups when compared with that in control (P<0.05 or P<0.01). This suggested that HBs exposure was able to induce release of cyt c from mitochondria to cytoplasm through the MPTP opening, and the higher the HBs concentration, the greater the amount of cyt c released.

Mitochondria are special energy-producing structures. Cells that are undergoing apoptosis or necrosis have more ADP compared to ATP because the cells are not producing ATP while hydrolyzing the remaining ATP into ADP. Thus, the changes in the ADP/ATP ratio have been used to differentiate the modes of cell death and viability. In the present study, the ADP/ATP ratios rose with increasing concentrations of HBs. There were significant differences in ADP/ATP ratios between control and all test groups (P<0.05, P<0.01). The levels of ADP, however, were still lower than ATP in the 100 µg/ml HBs-exposed group (0.83 vs 1.00), suggesting that only part of the mitochondria underwent opening of MPTP and the cell still had a sufficient level of ATP to direct cell death through the apoptotic pathway [18]. Thus, HBs led to spermatozoa apoptosis in this study.

In the present study, HBs exposure induced the early events in the apoptotic cascade. The induction of MPT by Ca^2+^ overload which increased mitochondrial membrane permeability, caused mitochondria to become further depolarized, meaning that MMP was abolished. This explains the mechanism through which HBs induced loss of MMP in our previous study [12]. Loss of MMP interferes with ATP production, because mitochondria must have an electrochemical gradient to provide the driving force for ATP production. Meanwhile, depolarization of mitochondria causes inhibition of oxidative phosphorylation and stimulation of ATP hydrolysis. That is why the ADP/ATP ratios rose with increasing concentrations of HBs in this study. When MMP is lost, protons and some molecules are able to flow across the outer mitochondrial membrane uninhibited [19], [20], leading to the release of apoptogenic proteins, such as cyt c, apoptosis-inducing factor and some pro-caspases, from the mitochondrial intermembrane space to cytoplasm [21], [17]. The released cyt c binds to apoptosis protease-activating factor 1 (Apaf-1) to form cyt c–Apaf-1 complex, which then activate caspase-9 [22], followed by activation of downstream death effectors such as caspase-3, caspase-6, and caspase-7 [23]. This can be used to explain our previous finding, in which HBs exposure was able to induce caspases-9, caspases-3 and caspases-8 activation in the spermatozoa [13].

However, an interesting question remains. How HBs triggers such responses in the spermatozoa? It has been demonstrated that the asialoglycoprotein receptor (ASGP-R) may play a role in the uptake of HBs into sperm cells [12]. The [Ca^2+^]_i_ was a signal initiating the process of apoptosis in this study. But it is still unknown how HBs induces Ca^2+^ overload after uptake into sperm cells, which should be further studied.

### Effects of HBs Exposure on Sperm Fertilizing Capacity

Sperm motility is the ability of sperm to move properly towards an oocyte. Hyperactivation, a type of sperm motility, can facilitate sperm progression towards the oocyte and penetration of its vestments [24]–[26]. In the present study, the average rate of MOT and the mean HA rate declined with increasing concentrations of HBs. There were significant differences in the average rates of MOT in 50 and 100 µg/ml HBs-exposed groups and in the mean HA rates in all test groups when compared with those in controls (P<0.05 or P<0.01). The sperm velocity also deceased with increasing concentrations of HBs. This suggested that HBs exposure could affect sperm motility, velocity and hyperactivation and in a dose dependent manner. AR is an exocytotic process of spermatozoa and an absolute requirement for fertilization [27]. ZP is the main physiological inducer of AR [27], and the ZPIAR will enhance the ability of sperm to penetrate the ZP and finally to fertilize oocytes [28]. In the present study, the average rates of ZPIAR were reduced with increasing concentrations of HBs. There were significant differences in the average rates of ZPIAR between control and all test groups (P<0.05; P<0.01). Therefore HBs exposure could decrease the rates of ZPIAR in spermatozoa and showed dose-dependent toxicity.

It has been documented that Ca^2+^ is a primary second messenger and triggers hyperactivated motility in spermatozoa [25], [29], and Ca^2+^ influx is an absolute requirement for physiological AR in spermatozoa [30]. In the present study, the [Ca^2+^]_i_ in spermatozoa rose markedly with increasing concentration of HBs. Accordingly HBs-induced [Ca^2+^]_i_ increase should result in elevating MOT, hyperactivation and ZPIAR in this study. Why were they, on the contrary, lowered? The following reasons may contribute to this discrepancy. Calcium is a two-faced messenger, with different calcium levels causing diverse responses. Normally, spermatozoa become physiologically loaded with calcium, causing hyperactivated motility and ZPIAR. After exposure to HBs, the spermatozoa became pathologically overloaded with calcium, causing mitochondrial dysfunction, including MPTP opening and a cascade of subsequent apoptotic events, leading to the following consequences. Firstly, energy is necessary for spermatozoa to maintain the function during capacitation, hyperactivation, acrosome reaction and fertilization [31], [32]. The mitochondrial depolarization caused loss of MMP, leading to inhibition of ATP production. The reduced energy supply was unable to meet the requirements for initiating and maintaining above functions. Next, the membrane integrity is closely related to sperm motility, capacitation, acrosome reaction and sperm-oocyte interactions [33]. Our previous findings have demonstrated that HBs exposure could induce ROS generation, lipid peroxidation and PS externalization in spermatozoa [13]. Such apoptotic events are able to cause loss of membrane integrity, leading to reduced rates of motility, hyperactivation and acrosome reaction. Furthermore, the induction of acrosome reaction involves plasma membrane receptors, intracellular signals and signal transduction pathways, and is mediated by an increase in [Ca^2+^]_i_. The calcium channels appear to be modulated either by plasma membrane potential or by protein phosphorylation [27]. All these involve integrity of sperm plasma membrane. When sperm loses membrane integrity, occurrence of acrosome reaction would be affected. Finally, the cyt c–Apaf-1 complex may play a role in lower sperm motility [34]. In fact, the opposing roles of calcium has been reported by some previous studies: on the one hand calcium is a key regulator of cell survival, on the other hand cellular Ca^2+^ overload, or perturbation of intracellular Ca^2+^ compartmentalization, can cause cytooxicity and trigger either apoptotic or necrotic cell death [35], [36].

In the present study, no significant difference in the number of sperms bound to ZP between control and test groups was found, suggesting that HBs exposure had no impact on binding of sperm to ZP.

In conclusion, exposure of HBs to human spermatozoa enhances the early events of apoptosis cascade and reduces sperm fertilizing capacity *in vitro*. This is an *in vitro* study, in which only mature and selected motile sperm were exposed to HBs. In the *in vivo* situation, sperms of a HBV-infected male will be exposed to the virus during spermatogenesis and afterwards. Both the testis and the epididymis will usually elicit an anti-viral immune response that should provide a certain level of protection absent in this *in vitro* situation. Thus, the *in vivo* impacts of HBs on male fertility require further investigation.

## Materials and Methods

### Ethical Approval

All healthy male donors and female patients, who were explicitly informed about the research aims, their rights and interests, signed consent forms permitting use of their gametes (sperm samples and unfertilized oocytes) for research. All the protocols used in the present study were approved by Institutional Ethical Review Board (IERB) of Shantou University Medical College (SUMC), and conformed to the ethical guidelines of the 1975 Declaration of Helsinki (The 6^th^ revision, 2008) as reflected in a priori approval by the institution’s human research committee.

### Preparations of Human Spermatozoa

Human sperm samples were obtained from healthy men by masturbation after 3 days of sexual abstinence. Semen samples were kept in a humidified incubator (37°C, 5% CO_2_ in air) for 30 min to allow liquefaction. Motile spermatozoa were selected by the swim-up method as follows: in each test tube, the 0.5 ml liquefied semen sample was layered gently under 2 ml of Biggers-Whittem-Whittingham (BWW) medium containing 0.3% bovine serum albumin (BSA) and incubated in a humidified incubator (37°C, 5% CO_2_ in air) for 1 h. The supernatant collected from tubes was centrifuged at 300 × g for 5 min, and the pellet of motile sperm was washed once. The final concentration of spermatozoa was adjusted to 1×10^6^ sperm/ml in BWW medium with 0.3% BSA for subsequent use.

### Exposure of Spermatozoa to HBs

Human spermatozoa were incubated in BWW medium with various concentrations of HBs (0, 25, 50, 100 µg/ml) (NCPC GencTech Biotechnology Co., LTD., Hebei, China) in a CO_2_ incubator (37°C, 5% CO_2_ in air) for 3 h, and then washed twice and adjusted to 1×10^6^ sperm/ml. The spermatozoa treated by HBs were used as test group, and the untreated spermatozoa were used as control group in the present study.

### Preparations of Human Oocytes

Oocytes which showed no evidence of two pronuclei or cleavage at 48–60 h after insemination by routine *in vitro* fertilization (IVF), or after injection by intracytoplasmic sperm injection (ICSI), or immature (germinal vesicle or metaphase I) oocytes not injected by ICSI were used for the sperm–ZP binding test. If the oocytes had sperms bound to the ZP from the IVF insemination, these were removed by aspiration using a fine glass pipette with an inner diameter (120 µm) slightly smaller than the oocyte diameter [37]. Oocytes with >10 sperms penetrating the ZP, or degenerated, activated or morphologically abnormal were not used. Oocyte samples were stored in 1 M ammonium sulphate solution at 4°C before use. The salt-stored oocytes required extensive washing with culture medium over a period of 4 h to remove the salt before incubation with sperm.

### Measurement of Changes in [Ca^2+^]**_i_**

The changes in the [Ca^2+^]_i_ were monitored with the fluorescent probe fluo-4 AM which is a high-affinity calcium indicator. The motile spermatozoa, prepared as mentioned above, were pre-incubated with 10 µM fluo-4 AM (Dojindo) containing 0.1% pluronic acid F-127 in a dark chamber in a humidified incubator (37°C, 5% CO_2_ in air) for 40 min followed by incubation with various concentrations of HBs (0, 25, 50, 100 µg/ml) for 3 h. The cells were washed twice and resuspended in 0.5 ml of phosphate buffered saline (PBS), and immediately analyzed by using flow cytometry (FCM) with laser excitation wavelength at 488 nm and emission filter 522DF35. In order to convert fluorescence values into absolute [Ca^2+^]_i_, the calibration was performed at the end of each experiment. [Ca^2+^]_i_ was calculated using the equation [Ca^2+^]_i_ = *K_d_* (*F* − *F*_min_)/(*F*_max_ − *F*), where *K_d_* is the dissociation constant of the Ca^2+^-fluo-4 complex, and *F* represents the fluorescence intensity of the cells. *F*_max_ represents the maximum fluorescence (obtained by treating cells with 20 µM calcium ionophore A23187), and *F*_min_ corresponds to the minimum fluorescence (obtained from ionophore-treated cells in the presence of 3 mM EGTA). The above experiment was repeated five times.

### Calcein/Co^2+^ Assay for MPTP Opening

Before exposure to HBs, the motile spermatozoa were loaded with Cal-AM (Dojindo, Kumamoto, Japan) to a final concentration of 15 µM in the presence of 30 µM CoCl_2_ (Sigma-Aldrich, UK) and 0.1% Pluronic F127 in a dark chamber in a humidified incubator (37°C, 5% CO_2_ in air). After 20 min incubation, the spermatozoa were washed twice with PBS to remove excess stain and quenching reagent, and then exposed to various concentrations of HBs (0, 25, 50, 100 µg/ml) for 3 h. The sperm samples were analyzed by using FACScan Flow Cytometer (BD biosciences, San Diego, CA, USA) equipped with a single 488-nm argon-ion excitation laser. The above experiment was repeated five times.

### FCM Analysis

FCM analyses were performed by using a FACScan Flow Cytometer (BD Biosciences, San Diego, CA). Cells were isolated from fragments by gating on the forward and side scatter signals, and then cells were identified and analyzed according to their relative fluorescence intensities compared with unstained cells. A minimum of 10,000 events were acquired and analyzed in each sample at the rate of 100–1000 events per second and data analysis was performed using BD Cell Quest and WinMDI 2.9. Results were expressed as mean fluorescence intensity.

### Assessment of Cyt c Release from Mitochondria

After exposure of spermatozoa (1×10^6^/ml) to various concentrations of HBs (0, 25, 50, 100 µg/ml) for 3 h, the preparation of cytosolic and mitochondrial fractions in spermatozoa were carried out by using Mitochondria/Cytosol Fractionation Kit (Catalog #K256, BioVision, CA, USA) according to the manufacturer’s instructions. Briefly, cells were washed with 10 ml of ice-cold PBS and centrifuged at 600×g for 5 min at 4°C. Supernatant was removed and cells resuspended with 1.0 ml of 1×Cytosol Extraction Buffer Mix containing DTT and protease inhibitors. Cells were incubated on ice for 10 min and homogenized in an ice-cold Dounce tissue grinder. After checking the efficiency of homogenization, homogenate was transferred to a 1.5-ml microcentrifuge tube, and centrifuged at 700×g for 10 min at 4°C. Supernatant was collected into a fresh 1.5-ml tube, and centrifuged at 10,000×g for 30 min at 4°C. This supernatant was stored as the cytosolic fraction. The pellet was resuspended in 0.1 ml Mitochondrial Extraction Buffer Mix containing DTT and protease inhibitors, vortexed for 10 s and saved as mitochondrial fraction.

The levels of cyt c in the cytosolic/mitochondria fractions were measured by using a Human Cytochrome c Quantikine ELISA Kit (R&D systems, Minneapolis, USA) according to the manufacturer’s instructions. ELISA was performed in triplicate (n = 3 per replicate) to calculate the average cyt c nanograms (ng) in the cytosolic or mitochondrial fractions per milligram (mg) of protein. The absorbance at 450 nm (a measure of cyt c concentration) was corrected by subtracting the background reading at 570 nm. The cyt c concentration was calculated by interpolating these values on a standard curve constructed for each plate. The above experiment was repeated five times.

### Measurement of the ADP:ATP Ratio

After exposure of spermatozoa (1×10^6^/ml) to various concentrations of HBs (0, 25, 50, 100 µg/ml) for 3 h, the measurement of the ADP:ATP ratio in spermatozoa was carried out by using an EnzyLight™ ADP/ATP Ratio Assay Kit (BioAssay Systems, USA) according to manufacturer’s instructions. Briefly, 10 µl of the washed spermatozoa (10^3^–10^4^) was transferred into a tube. 90 µl ATP Reagent was added to the tube and vortexed for 30 s. After 1 min, luminescence (RLU A) was recorded. Ten min later reading was repeated (RLU B). This measurement provided background prior to measuring ADP (i.e. the residual ATP signal). Immediately following reading RLU B, 5 µl ADP reagent was added to the tube and vortexed. After 1 min, luminescence (RLU C) was obtained. ADP/ATP Ratio = RLU C – RLU B/RLUA. All samples were performed in triplicate. The data from the ATP/ADP assay is presented as an average of the ATP/ADP ratios. The above experiment was repeated five times.

### Assessment of Sperm Motility Parameters

After exposure of spermatozoa (2×10^6^/ml) to various concentrations of HBs (0, 25, 50, 100 µg/ml) for 3 h, the MOT, HA, velocities (VSL, VCL, VAP) and other characteristics including CON, PMOT, STR, LIN, ALH and BCF were measured by using the Computer-Assisted Sperm Analyzer (Hamilton-Thorn Research, Danvers, MA, USA) at 37°C. The Burkman (1991) criteria for HA were used as follows: VCL≥100 um/s, LIN≤65% and amplitude of lateral head displacement (ALH)≥7.5 µm. Motility was defined as the percentage of sperm with VAP>7.5 µm/s. Sperm concentration was adjusted to ∼20×10^6^/ml by centrifugation at 700×g for 5 min and resuspended in 100 µl of the same medium. A sample of 5 µl was placed in a microcell (18.7 µm depth) for assessment of motility and velocities. For each sperm sample, an average of six (five to seven) fields with a total of 300–400 sperms was assessed.

### Sperm- ZP Binding Test

Sperm-ZP binding test was performed as described previously [28]. Briefly, after exposure of spermatozoa (2×10^6^/ml) to various concentrations of HBs (0, 25, 50, 100 µg/ml) for 3 h, motile sperms of each group were incubated with four oocytes in a humidified incubator (37°C, 5% CO_2_ in air) for 2 h, and then transferred to PBS containing 2 mg/ml bovine serum albumin (BSA, Sigma Chemicals Co., St. Louis, MO). The oocytes were flushed several times to dislodge loosely adherent sperm using a fine pipette approximately twice the diameter of the oocyte. The number of sperms bound to each of four oocytes was counted using an inverted phase contrast microscope and the average number of sperms bound per ZP was used as endpoint. Oocytes with>100 sperms bound were recorded as 100 since it is impossible to count the number accurately. In the present study, the above experiment was repeated five times and the average number of sperms bound to ZP in each group is expressed as median and quartile deviation per ZP.

### Assessment of the AR of Sperms Bound to the ZP

Assessment of the AR of sperms bound to the ZP was performed as described previously [28]. Briefly, after counting the number of sperms bound to the ZP, all sperms bound to the surface of the four ZPs were removed by repeated vigorous aspiration with a narrow gauge pipette with an inner diameter of about 120 mm. This was performed on a glass slide with ∼5 µl PBS containing 0.2% BSA and dislodged sperms were smeared in a limited area (∼16 mm^2^), which was marked on the back of the slides with a glass pen to help find the sperm under the microscope for assessment of the AR. The AR of dislodged ZP-bound sperm was assessed using Pisum sativum agglutinin (PSA) conjugated with fluorescein isothiocyanate (Sigma Chemical Company, St Louise, MO, USA) as described previously [38]. After air-drying, sperm smears were fixed in 95% ethanol for 30 min and then stained using 25 µg/ml PSA in PBS for 2 h at 4°C. The slides were washed and mounted with distilled water and 200 sperms per sample were counted with a fluorescence microscope using excitation wavelengths of 450–490 nm and a magnification of 400 x. When more than half the head of a sperm was brightly and uniformly fluorescent, the acrosome was considered intact. Sperm with a fluorescent band at the equatorial segment or without fluorescence (a rare pattern) were considered acrosome reacted.

### Statistical Analysis

The data are representatives of average of five independent experiments using sperm sample from five different men. The average values of data are expressed as mean ± SD. SPSS 17.0 programs were used in the statistical analysis. A paired t test was performed to evaluate the impact of HBs exposure. The number of sperms bound to ZP in each group is expressed as median and quartile deviation. A Kruskal-Wallis test in GraphPad Prism 5 was conducted to evaluate the differences among various concentrations of HBs (0, 25, 50, 100 µg/ml) on median change in number of sperms bound to ZP. P-value <0.05 was considered to be significant.

## References

[1] World Health Organization (WHO). Hepatitis B. Fact Sheet WHO/204 (2008) Revised October 2008.

[2] Ganem D (1996) Hepadnaviridae and their replication. In Fields BN, Knipe DM, Howley PM, editors. Fields Virology, 3^rd^ edn. Philadelphia: Lippincott-Raven. 2703–2738.

[3] Scott RM, Snitbhan R, Bancroft WH, Alter HJ, Tingpalapong M (1980) Experimental transmission of hepatitis B virus by semen and saliva. J Infect Dis 142: 67–71. 7400629 10.1093/infdis/142.1.67

[4] Hadchouel M, Scotto J, Huret JL, Molinie C, Villa E, et al (1985) Presence of HBV DNA in spermatozoa: a possible vertical transmission of HBV via the germ line. J Med Virol 16: 61–66. 3840197 10.1002/jmv.1890160109

[5] Lang ZW (1993) [Distribution of hepatitis B virus in testicle tissue in patients with hepatitis B infection]. Zhonghua Yi Xue Za Zhi 73: 329–331, 379. 8258099

[6] Huang JM, Huang TH, Qiu HY, Fang XW, Zhuang TG, et al (2002) Studies on the integration of hepatitis B virus DNA sequence in human sperm chromosomes. Asian J Androl 4: 209–212. 12364978

[7] Oger P, Yazbeck C, Gervais A, Dorphin B, Gout C, et al (2011) Adverse effects of hepatitis B virus on sperm motility and fertilization ability during IVF. Reprod Biomed Online 23: 207–212. 21665545 10.1016/j.rbmo.2011.04.008

[8] Bejarano I, Espino J, Paredes SD, Ortiz A, Lozano G, et al.. (2012) Apoptosis, ROS and Calcium Signaling in Human Spermatozoa: Relationship to Infertility. In Bashamboo A, McElreavey KD, editors. Male infertility. InTech-Open Access Publisher. 51–76.

[9] Richburg JH (2000) The relevance of spontaneous- and chemically-induced alterations in testicular germ cell apoptosis to toxicology. Toxicol Lett 112–113: 79–86. 10.1016/s0378-4274(99)00253-2 10720715

[10] Shen HM, Dai J, Chia SE, Lim A, Ong CN (2002) Detection of apoptotic alterations in sperm in subfertile patients and their correlations with sperm quality. Hum Reprod 17: 1266–1273. 11980750 10.1093/humrep/17.5.1266

[11] Moretti E, Federico MG, Giannerini V, Collodel G (2008) Sperm ultrastructure and meiotic segregation in a group of patients with chronic hepatitis B and C. Andrologia. 40: 286–291. 10.1111/j.1439-0272.2008.00855.x 18811918

[12] Zhou XL, Sun PN, Huang TH, Xie QD, Kang XJ, et al (2009) Effects of hepatitis B virus S protein on human sperm function. Hum Reprod 24: 1575–1583. 19279032 10.1093/humrep/dep050

[13] Kang X, Xie Q, Zhou X, Li F, Huang J, et al (2012) Effects of hepatitis B virus S protein exposure on sperm membrane integrity and functions. PLoS One 7: e33471. 22470450 10.1371/journal.pone.0033471 PMC3314651

[14] Paasch U, Grunewald S, Dathe S, Glander HJ (2004) Mitochondria of human spermatozoa are preferentially susceptible to apoptosis. Ann N Y Acad Sci 1030: 403–409. 15659823 10.1196/annals.1329.050

[15] Salisbury JJ, Bradford JA, Godfrey WL, Ignatius MJ, Janes MS. Relationships between mitochondrial permeability transition pore activity and mitochondrial membrane potential during apoptosis. Dec. 4–8, 2004; The American Society for Cell Biology 44th Annual Meeting, Washington, DC., USA.

[16] Bernardi P, Krauskopf A, Basso E, Petronilli V, Blachly-Dyson E, et al (2006) The mitochondrial permeability transition from in vitro artifact to disease target. FEBS J 273: 2077–2099. 16649987 10.1111/j.1742-4658.2006.05213.x

[17] Halestrap AP, McStay GP, Clarke SJ (2002) The permeability transition pore complex: another view. Biochimie 84: 153–166. 12022946 10.1016/s0300-9084(02)01375-5

[18] Javadov S, Karmazyn M, Escobales N (2009) Mitochondrial permeability transition pore opening as a promising therapeutic target in cardiac diseases. J Pharmacol Exp Ther 330: 670–678. 19509316 10.1124/jpet.109.153213

[19] Schinder AF, Olson EC, Spitzer NC, Montal M (1996) Mitochondrial dysfunction is a primary event in glutamate neurotoxicity. J Neurosci 16: 6125–6133. 8815895 10.1523/JNEUROSCI.16-19-06125.1996 PMC6579180

[20] White RJ, Reynolds IJ (1996) Mitochondrial depolarization in glutamate-stimulated neurons: an early signal specific to excitotoxin exposure. J Neurosci 16: 5688–5697. 8795624 10.1523/JNEUROSCI.16-18-05688.1996 PMC6578963

[21] Crompton M (1999) The mitochondrial permeability transition pore and its role in cell death. Biochem J 341 (Pt 2): 233–249. PMC1220352 10393078

[22] Zou H, Henzel WJ, Liu X, Lutschg A, Wang X (1997) Apaf-1, a human protein homologous to C. elegans CED-4, participates in cytochrome c-dependent activation of caspase-3. Cell 90: 405–413. 9267021 10.1016/s0092-8674(00)80501-2

[23] Thornberry NA, Lazebnik Y (1998) Caspases: enemies within. Science 281: 1312–1316. 9721091 10.1126/science.281.5381.1312

[24] Yanagimachi R (1994) Mammalian fertilization. In Knobil E, editor. The Physiology of Reproduction. New York: Raven Press. 189–317.

[25] Ho HC, Suarez SS (2001) An inositol 1,4,5-trisphosphate receptor-gated intracellular Ca(2+) store is involved in regulating sperm hyperactivated motility. Biol Reprod 65: 1606–1615. 11673282 10.1095/biolreprod65.5.1606

[26] Suarez SS, Pacey AA (2006) Sperm transport in the female reproductive tract. Hum Reprod Update 12: 23–37. 16272225 10.1093/humupd/dmi047

[27] Patrat C, Serres C, Jouannet P (2000) The acrosome reaction in human spermatozoa. Biol Cell 92: 255–266. 11043413 10.1016/s0248-4900(00)01072-8

[28] Liu DY, Liu ML, Clarke GN, Baker HW (2007) Hyperactivation of capacitated human sperm correlates with the zona pellucida-induced acrosome reaction of zona pellucida-bound sperm. Hum Reprod 22: 2632–2638. 17656416 10.1093/humrep/dem245

[29] Xia J, Reigada D, Mitchell CH, Ren D (2007) CATSPER channel-mediated Ca2+ entry into mouse sperm triggers a tail-to-head propagation. Biol Reprod 77: 551–559. 17554080 10.1095/biolreprod.107.061358

[30] Vasudevan SR, Lewis AM, Chan JW, Machin CL, Sinha D, et al (2010) The calcium-mobilizing messenger nicotinic acid adenine dinucleotide phosphate participates in sperm activation by mediating the acrosome reaction. J Biol Chem 285: 18262–18269. 20400502 10.1074/jbc.M109.087858 PMC2881750

[31] Vandevoort CA, Overstreet JW (1995) Effects of glucose and other energy substrates on the hyperactivated motility of macaque sperm and the zona pellucida-induced acrosome reaction. J Androl 16: 327–333. 8537250

[32] Misro MM, Ramya T (2012) Fuel/Energy Sources of spermatozoa. In Parekattil SJ, Agarwal A, editors. Male Infertility. New York, Heidelberg, Dordrecht, London: Springer. 209–211.

[33] Cross NL, Hanks SE (1991) Effects of cryopreservation on human sperm acrosomes. Hum Reprod 6: 1279–1283. 1752931 10.1093/oxfordjournals.humrep.a137526

[34] Said TM, Paasch U, Glander HJ, Agarwal A (2004) Role of caspases in male infertility. Hum Reprod Update 10: 39–51. 15005463 10.1093/humupd/dmh003

[35] Orrenius S, Zhivotovsky B, Nicotera P (2003) Regulation of cell death: the calcium-apoptosis link. Nat Rev Mol Cell Biol 4: 552–565. 12838338 10.1038/nrm1150

[36] Demaurex N, Distelhorst C (2003) Cell biology. Apoptosis–the calcium connection. Science 300: 65–67. 12677047 10.1126/science.1083628

[37] Liu DY, Baker HW (2004) High frequency of defective sperm-zona pellucida interaction in oligozoospermic infertile men. Hum Reprod 19: 228–233. 14747159 10.1093/humrep/deh067

[38] Liu DY, Baker HW (1996) A simple method for assessment of the human acrosome reaction of spermatozoa bound to the zona pellucida: lack of relationship with ionophore A23187-induced acrosome reaction. Hum Reprod 11: 551–557. 8671264 10.1093/humrep/11.3.551

